# Exploration of facilitators and barriers to the implementation of a guideline to reduce HIV-related stigma and discrimination in the Ethiopian healthcare settings: A descriptive qualitative study

**DOI:** 10.1371/journal.pone.0216887

**Published:** 2019-05-13

**Authors:** Garumma Tolu Feyissa, Mirkuzie Woldie, Zachary Munn, Craig Lockwood

**Affiliations:** 1 Jimma University, Department of Health, Behavior and Society, Jimma University, Jimma, Ethiopia; 2 Ethiopian Evidence Based Health Care Centre: JBI Center of Excellence, Jimma University, Jimma, Ethiopia; 3 The Joanna Briggs Institute, the University of Adelaide, Adelaide, South Australia, Australia; 4 Department of Health Policy and Management, Jimma University, Jimma, Ethiopia; 5 Department of Global Health and Population, T.H. Chan Harvard School of Public Health, Addis Ababa, Ethiopia; University of Notre Dame Australia, AUSTRALIA

## Abstract

**Background:**

The barriers to uptake of guidelines underscore the importance of going beyound the mere synthesis of evidence to tailoring the synthesized evidence into local contexts and situations. This requires in-depth exploration of local factors. This project aimed to assess contextual barriers and facilitators to the implementation of a guideline developed to reduce HIV-related stigma and discrimination (SAD) in the Ethiopian healthcare setting.

**Methods:**

A descriptive qualitative research study was conducted using a semi-structured interview guide informed by the Registered Nurses Association of Ontario (RNAO) framework. The interview was conducted among a purposive sample of seven key informants from Jimma University and Jimma Zone HIV Prevention and Control Office. The interviews were transcribed, coded and analysed using Atlas ti version 7.5 software packages.

**Results:**

Guideline attributes, provider-related factors and organizational and practice-related were identified as factors that can potentially affect the implementation of the guideline. The presence of expert patients were identified as agents for guideline implementation, whilst regular health education programs in addition to initiatives related to service quality improvement, were identified as suitable platforms to assist with the implementation of this guideline. Study participants recommended that the guideline should be disseminated through multidisciplinary team (MDT) meetings, gate keepers such as opinion leaders and unit heads, one-to-five networks and mentorship programs, as well as training, workshops and posters. The current study also indicated that continuous monitoring, evaluation and mentorship are critical elements in the integration of the guideline into the system of the hospital.

**Conclusions:**

This study identified that guideline implementation can make use of existing structures and pathways such as MDT meetings, service quality improvement initiatives, one-to-five networks, training and workshops. Teamwork and partnership with stakeholders should be strengthened to strengthen facilitators and tackle barriers related to the implementation of the guideline. Effective implementation of the guideline also requires establishing an implementation structure. Moreover, indicators developed to track the implementation of stigma reduction guideline should be integrated into mentorship, MDT meetings and evaluation programs of the hospital to improve performance and to assist data collection on implementation experiences.

## Background

Ethiopia is one of the 20 countries contributing to 80% of the global burden of the human immunodeficiency virus (HIV); in 2018, there were- an estimated 610,000 people living with HIV (PLHIV) in Ethiopia which makes up an adult prevalence rate of 0.06%. The annual incidence of HIV among adults aged 15 to 64 years in urban Ethiopia is 0.06%. This corresponded to 7,000 new cases of HIV annually among urban adults aged 15 to 64 years living in Ethiopia [1]. Stigma and discrimination (SAD) related to HIV have deterred HIV prevention and control activities in the country [2]. Reduction of HIV-related SAD is one of the priority targets of the Federal Ministry of Health (FMOH) of Ethiopia, regional state health bureaus and HIV prevention and control offices at different levels [3].

The country’s progress report of 2014 on HIV response shows that stigma remains a significant challenge and obstacle towards the effectiveness of HIV prevention and control [3]. The 2018 United Nations (UN) data indicated that nearly 50% of adults reported discriminatory attitudes towards PLHIV [4]. Other studies indicate the persistence of HIV-related stigma among rural and urban communities [5, 6], key population groups [7], and among health care providers [8]. Stigma appears to cross all geographic and social boundaries in Ethiopia despite the widespread burden of disease across the community [9].

Stigma and discrimination are associated with poor psychosocial adaptation among PLHIV [10]. Because of the expansion of antiretroviral therapy (ART), the survival rate among PLHIV has increased. The fact that more PLHIV are living longer than before implies that more people are in need of psychosocial support for better psychosocial adaptation [11]. Furthermore, as part of the comprehensive needs of PLHIV, home-based care activities and pharmaco-therapeutic adherence support programs are often run by volunteers and patients in Ethiopia [12]. However, in the presence of SAD, support programs and other activities run by volunteers are less likely to be successful. Being visited by volunteers is associated with the experience of stigma. The fear of stigma results in reduced rate of attendance of care delivered by volunteers [13]. Problematically, there are different manuals and guidelines on prevention, care and support related to HIV that are in use in Ethiopia, causing fragmentation of care [14]. Although no guidelines specifically address SAD in healthcare settings, all the manuals acknowledge the impact of SAD [14].

The Joanna Briggs Institute (JBI) model of evidence-based healthcare encourages the consideration of local context in the implementation of evidence-based practices [15]. The existence of synthesized or summarized evidence in the form of systematic reviews and guidelines by itself is not enough for the improvement of policy and practice [15, 16].

Different factors that impede the implementation of guidelines have been reported. These include: resource related barriers, difficulty of understanding the recommendations, the lack of awareness of the existence of the guidelines by healthcare workers (HCWs), the lack of management support and work overload [17, 18]. These barriers to uptake underscore the importance of going beyond the mere synthesis of evidence to tailoring the synthesized evidence into local contexts and situations [15, 19]. The impact of these barriers on guideline uptake may vary across different contexts and localities [20].

A multidisciplinary team of experts have developed a guideline to reduce stigma and discrimination based on evidence generated from systematic reviews [21, 22]. A consensus on the guideline recommendations was established through evaluation by experts using a series of Delphi surveys [23]. Before implementing the guideline in the Ethiopian healthcare settings, local factors that affect the implementability of the guideline need to be identified and should be considered while planning the implementation of a guideline.

This project was aimed to assess potential barriers and facilitators to the implementation of the guideline and identify tailored recommended activities to maximize the uptake of the guideline. This study was part of a larger project that involved a systematic search and the development, evaluation and implementation ofthis guideline reviews [21–23].

Specifically the project aimed:

1. To identify the potential barriers to the implementation of the SAD reduction guideline.
2. To determine tailored solutions for the expected barriers to the implementation of the SAD reduction guideline.
3. To identify the potential facilitators for the implementation of the SAD reduction guideline.
4. To identify strategies for the implementation of the guideline for better uptake and adherence.

## Methods

### Ethical approval and consent to participate

The entire research project received ethical approval from the Human Research Ethics Committee of the University of Adelaide (approval number H-2016-140) and from the Institutional Review Boardof Jimma University Institute of Health (RPGC/389/2016). Written consent forms were obtained before commencing the interviews with the key informants. During the write-up and report writing, participants were not identified by their names, positions or roles; only codes were used.

### Research design and setting

A descriptive qualitative study was carried out between August and December, 2016 in Jimma Medical Centre (JMC). JMC is part of Jimma University, located in Jimma, a town 352kms southwest of Addis Ababa, the capital of Ethiopia. The medical center provides HIV-related services through its HIV and Tuberculosis Clinic and the Jimma University HIV Prevention and Control Office (JUHAPCO). The JMC provides inpatient and outpatient health services for a catchment population of more than 20 million people living in Southwest Ethiopia and neighboring countries such as South Sudan [24].

The JUHAPCO implements comprehensive HIV prevention activities including educational programs, outreach services, training and care and support for groups affected by HIV in Jimma and the surrounding community. It also provides technical support, training and mentorship for the HIV and Tuberculosis Care Clinic and other healthcare facilities in Jimma Zone, and learning resources for students, staff and researchers [25].

We conducted this project using a collaborative model with study participants to obtain information to tailor a stigma reduction guideline. The principal investigator (GTF) led all phases of the project (research design, data collection, analysis, interpretation and write up). While exploring information from the perspective of the end users of the guideline, GTF acted as a facilitator to elicit and record thoughts and beliefs of the guideline’s end users about factors that would positively or negatively impact the implementation of the guideline.

### Study population

A purposive sample of seven health professionals and health managers from Jimma University, JMC, JUHAPCO, and Jimma Zone HIV Prevention and Control Office participated in the interviews as key informants. The participants were selected based on their current roles as clinicians, mentors, trainers and managers related to HIV prevention and control.

### Data collection

Data were collected using a semi-structured interview guide (S1 Doc), adapted from the Registered Nurses Association of Ontario (RNAO) [26]. Based on this framework, the expected barriers and facilitators to the implementation of best practice guidelines are generally categorized into: evidence related factors, target audience related factors, resources needed for the implementation, and organizational context in which the guideline is to be implemented [26]. The guide was translated into local languages (Afan Oromo and Amharic) by the principal investigator (PI) and translated back into English by another person fluent in the languages to check semantic equivalence.

The key informants were provided with the copy of the stigma reduction guideline prior to the interview. Interviews were conducted by GTF, who digitally recorded all the interviews and took notes to supplement the analysis with gustures and facial expressions. Each interview was conducted at a place and time that was suitable for the participant. The initial findings of the interviews were further explored, and the opinions of the participants were cross-compared.

### Data processing and analyses

The recordings of the interviews were transcribed verbatim and translated into English. The transcripts were coded and analyzed using Atlas.ti 7.5 software package for qualitative data analyses. The data were analyzed thematically drawing on the framework suggested by Braun and Clark [27]. The transcripts were repeatedly read to achieve immersion and obtain the sense of the whole data. Then, data were systematically coded. The codes were then gathered in themes or patterned responses. After that, findings were organized thematically based on replication (confirming what other participants have said), extension (providing additional contextual information that extends findings) and refutation (providing a contrary view to what other participants said). Finally, the themes were reviewed and defined to generate the final report.

### Data quality control

To establish trustworthiness we employed the following mesures in this project. First, we adopted a well-established semi-structured interview guide to clearly incorporate the concepts under study. Secondly, we sought information from pariticipants with relevant expertise and experience [28]. Thirdly, data were collected by a researcher who was already familiar withthe culture of the local organization under study and who in the current research assumed a neutral role [28]. Fourthly, we attempted to verify the viewpoints of different experts against those of others through constantly reviewing the list of questions and further probing to get details of variations (negative case analyses) and including the opinions of all the participants [29]. Fifthly, we selected participants with relevant expertise in the field to enhance the dependability our findings [30].

Confirmability is achieved by the collection of thick descriptive data, negative case analyses and arranging for a confirmability audit and establishing referential adequacy [31]. In this project, accurate records of the responses of the participants were made during the interviews. In addition, unique opinions were further explored to understand how and why they disagreed with the more popular opinions. This was done through preliminary analysis of the data and through revising note books and modifying a list of questions based on the emerging themes.

The transferability of qualitative evidence is based on the similarity of contextual factors in the settings [29]. The potential transferability of the evidence to other settings should be considered in view of the procedures, settings and context described in this project. Moreover, the data generated in this project by itself is predominantly the description of the contextual situation in JMC from the perspective of factors related to guideline implementation.

### Operational definitions

#### One-to-five network

An ad hoc structure existing in most public organizations in Ethiopia where a team of five to six work colleagues regularly (usually every week) meet to discuss planned activities, implementation challenges and to propose future plans depending on contextual factors.

#### Expert patients

‘HIV positive lay health workers who function as adherence counsellors, health educators, outreach workers and often community advocates for other patients living with HIV.’[32]^(pp.3)^

#### Healthcare setting

Any type of healthcare facility, which may include, but not limited to hospitals, health centers, clinics and health posts.

#### Healthcare workers

Any health personnel, regardless of their year of training or whether they have had specialty training or not, who are involved in the provision of professional healthcare for patients in health facilities. These may include, but not limited to health professionals from different disciplines including, Nurses, Medical Doctors, Laboratory Technicians, Medical Anthropologists, Medical Sociologists, Psychologists and Psychiatrists, Health Promotion experts, Midwives, Pharmacists, Health Extension Workers and community volunteers.

#### HIV-related stigma

Is defined as ‘prejudice, discounting, discrediting and discrimination directed at people perceived to have HIV or AIDS and individuals, groups and communities with which they are associated.’

#### Barriers and facilitators

*F*actors are considered as facilitators if their presence promotes the implementation of, or adherence to the guideline. Factors are considered as barriers if they impede implementation of, or adherence to the guideline. The same factor can be both a barrier and a facilitator. If the presence of a factor was a facilitator, its absence was considered as a barrier.

## Results

A total of seven key informants participated in the interviews. The disciplinary backgrounds of the participants were medical doctors, nurses, midwives, medical officers and health promotion experts with further specialty training in clinical and public health disciplines.

Using open-coding technique, 119 codes emerged, which were then categorized under 97 categories that were further grouped under 32 subthemes. The subthemes were finally grouped under eight broader themes. Results were presented and described based on the following broad themes: barriers and facilitators, dissemination approaches, training, implementation, monitoring and evaluation (M&E), resource implications, integration of the guideline into the hospital routine, sustainability and scaling-up.

### 1.1 Theme 1: Barriers and facilitators to the implementation of the guideline

The barriers and facilitators identified through the interviews were disintegrated into three domains: characteristics of the guideline, existing opportunities and platforms in the policy and practice environment, and provider-related factors (Table 1). Most of the factors described can be both facilitators and barriers. If their absence is a barrier, their presence can be a facilitator for the implementation of the guideline. So, we did not want to make a demarcation between the two (between barriers and facilitators) while presenting them.

**Table 1:** Barriers and facilitators to the implementation of HIV-related stigma and discrimination reduction guideline.

Sub-themes	Categories	Subcategories	Codes
Characteristics of the guideline	Addressing gap in evidence and practice	The persistence of stigma	Stigma historically overlooked
			Stigma common among clients
			Stigma widely observed among HCWs
		Addressing stigma as a priority problem	Absence of guideline
			Gaps in handling clients
			Deviation from standard practice
		Supporting recommendations by global evidence	Recommendations developed based on systematic literature search and panel consensus
	Clarifying the scope of the guideline	Specifying the target users	Relating the guideline to specific jobs of HCWs
		Suggested format for different disciplines	Same format versus different format
			Integration of guidelines
			Enable HCWs to identify their roles and responsibilities
		Specifying the roles of other stakeholders	
	Comprehensiveness, clarity and consistency of the recommendations	Description of methods used to develop recommendations	
		Clarity of recommendations	
		Comprehensiveness of the guideline	
		Balance between clarity and comprehensiveness	
	Addressing ethical principles and issues related to patient charter	Having common goals with good governance	
		Addressing issues related to patient charter	
		Mentioning the rights and roles of patients	Services that clients should receive
			Service environment
	Making the guideline appealing and attractive	Preparing the guideline in the form of posters	
	Indication of steps required for the implementation	Description of where and how to start implementation	Deciding the unit in which to start
			Description of the steps in the implementation
	The presence of implementation tools	Mentorship tools	
		Evaluation tools	PLHIV-friendly health facility checklist
			HCW questionnaires
Organizational policy and practice related factors	Commitment of stakeholders	Commitment of hospital management	
		Presence of stakeholders that support HIV programs	
		Stigma reduction as priority of stakeholders	
		HIV as a focus area of policy makers	
		JMC is a favourable environment	
		Commitment of HCWs	
		Commitment of funders/partners	
	Existing agents and programs asopportunities	Expert patients	
		Associations of PLHIV	
		Regular health education programs	
		Mentorship programs	
		MDT meeting	
		One-to-five networks	
	Complementarities with existing programs	Addressing stigma as a roadway to achieve priority goals	Adherence to ART
			PMTCT utilization
			Zero new HIV infections
	Complementarities with new programs, initiatives and movements	The CRC initiative	
		Quality movement	
		Emphasis given for good governance	
		CASH	
	Patient load	Potential long-term effect on patient load	Stigma reduction leading to the reduction of patient load in the long-run
		Potential short-term effect on patient load	Implementation as potential time consumer
		High patient load impedes guideline implementation	
Provider-related factors	Knowledge and attitude of HCWs	Limited awareness of the guideline	If HCWs are not aware of the guideline, they will not be able to implement it.
		The perception that the guideline is imposed on them	
		Unrealistic expectations	Expecting incentives to attend training and to implement the guideline
		Failure of HCW’s to recognize and acknowledge their stigmatizing behaviours	The perception that they do not stigmatize and do not need a guideline
	HCWs being occupied by other competing interests		
	Motivation and commitment	Motivation of staff working in HIV and TB clinic	
		Presence of motivated staff to provide training	
	Sense of ownership of the guideline	Sense of ownership because of involvement during development	Involvement of professionals from local institution
		Sense of ownership during implementation	Perception that the implementation of the guideline is the responsibility of those individuals who received the initial training

**NB**: HCWs: Healthcare workers, HIV: Human immunodeficiency virus, PLHIV: People Living with HIV, JMC: Jimma Medical Centre, MDT: Multidisciplinary team, ART: Antiretroviral therapy, PMTCT: Prevention of Mother to Child Transmission, CASH: Clean and safe health facility, CRC: Compasionate, respectful and caring, TB: Tuberculosis.

#### 1.1.1 Characteristics of the guideline

The following factors inherent to the guideline were identified as facilitators for the implementation of the current guideline: addressing a gap in evidence and practice; comprehensiveness, clarity and consistency of recommendations; addressing ethical principles and issues related to patient charter; clarifying the scope of the guideline; indication of the steps required for the implementation; the presence of implementation tools; and making the guideline appealing and attractive.

#### Addressing a gap in evidence and practice

The first facilitator inherent to the guideline was the fact that the guideline addresses gaps in evidence and practice. Key informants pointed out that there is a practice gap in addressing stigma. They described this gap as an opportunity for better uptake of the guideline and better support for the guideline by different stakeholders. This is because addressing SAD, which is one of the priority problems, is a precondition to achieve HIV-related goals. They also reported that SAD related to HIV have been overlooked in the past relative to the focus given to the medical therapy of HIV. Mentioning that SAD related to HIV and its impacts are widely observed among clients and providers, participants also reported that the current prevailing gaps in handling clients is attributed to the lack of guidelines. The presence of these gaps placed an increased demand for the new guideline to reduce HIV-related SAD increasing the likelihood ofits implementation.

“As a guideline addressing our current gaps, there are opportunities that enhance the uptake of the guideline. People from nearby communities go somewhere else to get HIV-related services. This is because, the clinic [TB and HIV Clinic] is already separated from other units of the hospital and clients are afraid of going there. Because, if they go there, by default, it will be clear that they are HIV positive. The clinic should have been part of the other units in the hospital. This did not happen because there was no guideline and there was no one concerned about the rights of the clients. If the guideline is implemented, managers will understand the problem. And this may result in full integration of HIV services into other hospital services.”

(KI P5)

#### Comprehensiveness, clarity and consistency of recommendations

Study participants reported that the guideline was clear and, at the same time, it had detailed information related to methodological issues in the guideline development.

“The recommendations are very clear. For any guideline for better implementation, there is a need to keep the balance between the burden in reading details and the clarity and completeness of the recommendations. If descriptions do not exist, sometimes it is difficult to understand. So, keeping the balance is the key. This was addressed in this guideline. The guideline recommendations are clear and short.”

(KI P7)

The clarification of the scope of the guideline was also mentioned as a precondition for the successful implementation of the guideline. This includes specifying the target users of the guideline and the roles of other stakeholders.

#### Addressing ethical principles and issues related to patient charter

Participants reported that current focus areas such as issues of patient charters and patient rights are potential opportunities that could facilitate the implementation of the current guideline. Participants mentioned that as one of the guidelines addressing the rights and responsibilities of patients and other ethical principles, there is an opportunity for better uptake of the current guideline. Therefore, by addressing ethical principles, the guideline will potentially complement the current priority areas and this will potentially increase the uptake of the guideline.

“The issue of governance and patient’s rights are always neglected by healthcare workers. In the future, however, this negligence cannot be tolerated anymore. So, we must work on it. Patients are asking for their rights. The government is also giving priority for these areas. Therefore, this guideline came at the right time and there are many opportunities for the implementation.”

(KI P4)

Other factors inherent to the guideline that are expected to facilitate the implementation of the guideline were indicating steps to launch the guideline, the presence of implementation tools and the attractiveness of the guideline. While the presence of implementation tools such as ‘PLHIV-friendly healthcare facilities’ and the healthcare workers’ questionnaires were raised as facilitators for the implementation of the stigma reduction guideline, participants recommended preparing guidelines in an attractive way, such as in the form of posters.

“The guideline contains clear steps and checklists that give us clear direction about the implementation. I think these steps are practical for our hospital. For instance, it indicates the importance of establishing a committee, assessing the setup and other essential steps. Therefore, these steps and checklists included in the guideline are very essential. They indicate clear direction. In our facility, we have limited guidelines and checklists like this guideline. This has created confusion and lack of consistency in practice. So, whether people come and go, work will be done based on checklists and steps provided in the guideline.”

(KI P5)

### 1.2 Organizational policy and practice related factors (the practice setting)

Participants identified existing opportunities that could facilitate the implementation of the guideline. These are: the commitment of stakeholders, existing opportunities and the complementarities of institutional and programmatic goals with the guideline goals. On the other hand, high patient load is expected to impede the implementation of the guideline.

#### The commitment of stakeholder

Participants reported that the commitment of stakeholders at multiple levels is required for the implementation of a guideline. They stated that the commitment of the hospital and stakeholders at national level is evidenced in their programmatic and institutional goals. Reducing SAD is also one of the priorities of JMC and the government of Ethiopia. The SAD reduction guideline, as stated by the participants, complements the hospital service quality improvement initiatives currently underway, which will potentially increase the commitment of the JMC.

“Even at national level, there are programs such as health sector transformation plan that support such [stigma reduction] initiatives. For instance, one of the targets at national level is to reduce new HIV infections by 90%. Focus was given for HIV prevention and control. So, they are committed to reduce stigma. The current guideline addresses stigma and discrimination, one of the areas of HIV prevention and control activities where significant gaps exist. Therefore, this guideline plays a significant role in the programs.”

(KI P2)

#### Existing agents and programs as opportunities

Factors that the participants mentioned as opportunities for the implementation of the current guideline were: the existence of expert patients and associations of PLHIV, the existence of regular health education programs, the existence of mentorship programs and multidisciplinary team (MDT) meetings.

The involvement of expert patients (HIV positive lay health workers) in SAD reduction was identified as one of the critical and practical recommendations. This is because expert patients are better informed and have witnessed or experienced SAD. In addition, as one of the issues of governance and accountability, participants recommended that expert patients should be involved in decision-making activities, such as being members of hospital committees and boards. The involvement of the associations of PLHIV was raised by the participants as one of the potential opportunities for the introduction of the guideline. This can be realized through training the members of the associations, informing them about their rights and responsibilities so that they will ask for their rights. If the members are informed about the guideline, they can track its implementation by claiming their rights and responsibilities wherever necessary. Pariticipants also reported that the existing regular health education program in JMC may be used as a platform to introduce the current guideline.

#### Complementarities with institutional and programmatic goals

Study participants reported that there are institutional and programmatic goals that need the reduction of SAD as a focus. These are already existing programs such as, programs to increase treatment uptake and antiretroviral therapy (ART) adherence, and prevention of mother to child transmission (PMTCT) service utilization. The achievement of the goals of these programs needs stigma reduction as an input. Additionally, participants reported that most of the current priority initiatives such as Clean and Safe Health Facility (CASH), quality movement, and Compassionate, Respectful, and Caring (CRC) by the Federal Minstry of Health (FMOH) could well be complemented by the intents of the current guideline.

For instance, the main purpose of the CRC initiative, as participants stated, is to make health professionals demonstrate compassion and respect towards their clients. These objectives were reported to align with those of the guideline on SAD reduction. The reduction of stigmatizing attitudes and actions towards clients regardless of their disease status, could help HCWs to develop compassionate, respectful and caring attitude towards their clients. Therefore, the guideline is expected to complement the achievement of the goals of the CRC initiative by contributing to the effort of changing the attitudes of health professionals. Conversely, the achievement of the goals of the CRC initiative could contribute to the success of SAD reduction programs. Therefore, the CRC initiative could be taken as an opportunity for the implementation of the current guideline.

The other area of complementarity reported was ‘quality service movement’ and ‘good governance’. The ‘quality service movement’ initiative encourages the delivery of patient-centered care. Participants reported that the SAD reduction guideline complements this quality service movement by contributing to the delivery of a more patient-centered care while avoiding stigmatizing actions during care. The good governance initiative encourages the engagement of PLHIV and the associations of PLHIV in decision-making issues related to care. This is again an opportunity as client engagement is part of the recommendations included in the current stigma reduction guideline.

Thirdly, infection prevention and patient safety is raised as one of the priority problems of JMC. The current guideline is expected to reduce extra precautions and encourage standard precautions among HCWs while providing care to PLHIV. If the guideline is implemented, it will reduce over utilization of protective equipment and materials and thereby saving resources. These resources could then be utilized only when they are needed. As mentioned by the participants, HCWs are currently utilizing extra precaution because of irrational fear of transmission and the absence of a guideline related to SAD.

“When staff are assigned to work in the clinic [HIV and TB clinic], they proceed to work without sufficient orientation. Except the recent progress being made, there are no adequate reading materials and library services through which they [healthcare workers] improve their practice. So, they [healthcare workers] think as if the virus jumps from the client to the provider. Sometimes, there is a time where they [HCWs] are afraid of greeting them [PLHIV]. If HCWs see something, even patient’s saliva on their shoes, they always bleach their shoes. Others unnecessarily wear masks or gloves, sometimes double gloves. This is because they think that HIV positive clients are thought to transmit tuberculosis and HIV all the time. The toilets are separately locked for the staff, and clients cannot use them. We observe significant extra precaution [while providing care to PLHIV] in our hospital.”

(KI P2)

Participants stated that adherence to standard precautions helps to reduce unnecessary wastage of resources and substandard practices by encouraging health professionals undergo uniform practices for all types of clients. Participants also reported that currently clients are expected to be on ART earlier than before. However, most clients are delaying from seeking treatment because of fear of stigma. Therefore, addressing SAD is critical to improve uptake of HIV testing and care-seeking behavior of clients. As participants reported, these factors would create the need for the current guideline and therefore, provide a favorable situation for the implementation of the stigma reduction guideline.

A critical potential barrier reported to negatively affect implementation of the current guideline was high patient load. As reported by study participants, the implementation of the guideline implies that HCWs should pay greater attention to the needs of clients which may mean spending more time with each patient. However, the presence of high patient load may reduce the time spent by the HCWs with each patient and hence impede the proper implementation of the guideline. In the long term, nonethless, the implementation of this guideline is expected to reduce patient load as clients will be getting treatment from their right locality if stigma is reduced.

“Because of the fear of stigma and discrimination, clients from Jimma town go and seek treatment from healthcare facilities in other towns. And clients from other areas come and receive treatment from [facilities in] Jimma. Even some either avoid getting tested or seeking treatment. Therefore, if we could reduce stigma and discrimination, we can also reduce unnecessary patient loads, because clients will be able to get services from nearby facilities.”

(KI P5)

### 1.3 Provider-related factors (attributes of health professionals)

Facilitators and barries in this category relate to healthcare workers’ knowledge, awareness and attitude, being occupied by other competing interests, HCWs’ motivation and the sense of ownership of the guideline. Unrealistic expectations and limited awareness about the guideline among HCWs were among the potential barriers reported. On the other hand, it was pointed that HCWs may not recognize and acknowledge their stigmatizing behaviors which potentially hinders the implementation of the current SAD reduction guideline.

“There are some professionals who stigmatize HIV. Some of them still do not attend the delivery of a mother who is HIV positive. But, they deny their stigmatizing behaviors. So, it is essential to give them orientation and training. If HCWs do not know how to discharge their responsibilities, they will carry out substandard activities. HCWs may perceive that they are not stigmatizing HIV positive clients and they do not need the guideline. But, we may convince them that the guideline is for every HCW not just for those HCW’s who stigmatize PLHIV. So, we must raise the awareness of HCWs.”

(KI P6)

As elaborated by study participants, unrealistic expectation of HCWs is another potential barrier to be faced during the implementation. Some HCWs expect incentives during training and at times, during implementation. On the other hand, it was noted that some professionals may perceive that the guideline is imposed on them as a commandment. As reported by study participants, this may result because of inadequate awareness or because they are not convinced about the initiative against stigma and discrimination by health workers. The presence of staff that is available and committed to provide training and services related to HIV was raised as a facilitating factor for the implementation of the guideline.

Though the impact was claimed to be minimal for the stigma reduction guideline, participants reported that the implementation of a new guideline may require HCWs to give more time and attention to clients than before. Therefore, as pointed out by the participants, it is expected that some professionals may be resistant to the changes needed. Nevertheless, these expected challenges are minimal and HCWs can be convinced of the potential benefit of the guideline.

Participants reported that the sense of ownership for the guideline is one of the key factors that influence the implementation of the guideline. They stated that guidelines and initiatives usually fail because of the lack of sense of ownership and they stressed the necessity of working to increase the sense of ownership among HCWs. In addition, they reported that the sense of ownership developed because of the involvement of professionals from the local institution is one of the facilitators for the successful implementation of the current guideline. In addition, participants reported that the consideration of local factors during the development would potentially increase its implementability.

Participants also stated that the implementation of other guidelines introduced earlier failed because it was perceived as the responsibility of only those individuals who received the initial training on the guideline. Participants stressed that the sense of ownership should be built even during the implementation of the guideline.

“Sense of ownership should be built even during the implementation of the guideline. From our experiences, what we have learnt is professionals feel that the initiative is only the concern of those individuals who have been trained on the topic of interest. For instance, regarding CASH initiative, we trained two to three professionals from each unit. The objective was that these trained workers will orient the remaining staff in their units. Nevertheless, in our case, many staff members perceived that such new practices or initiatives are only the business and concern of those trained individuals.”

(KI P1)

The barriers and facilitators for the current guideline are summarized in Table 1.

### 2.1 Theme 2: Dissemination approaches

As a means of effective guideline implementation, the need for well planned dissemination was emphasized. The dissemination strategies identified were categorized into passive and active dissemination strategies. Traditional dissemination strategies such as official letters, publishing the guideline, distributing hard copies and availing the guideline in libraries and websites were identified as passive methods of dissemination. On the other hand, training, short term workshops, peer education, using unit heads as gate keepers, posters and media were identified as active strategies for dissemination. Moreover, study participants reported that the mentorship, MDT meetings and one-to-five networks that exist in the Ethiopian healthcare system may be utilized as active dissemination platforms.

“There were times when we introduced guidelines passively through official letters and distributing hard copies. But this was not effective. However, there was a time when we were effective in introducing the guidelines through active methods such as MDT meetings, through our mentorship and training programs.”

(KI P5)

The first strategy suggested for the dissemination of the current guideline was the MDT meeting, a session in which HCWs working on different areas related to HIV discuss issues related to their practices in the care and support for PLHIV, including their strengths, weaknesses and the challenges faced at work. This was suggested as a platform for the dissemination and implementation of the current guideline.

The second potential strategy for the dissemination of the guideline is mentorship, which is an onsite training where experienced health professionals teach other junior and less experienced professionals. The third potential disseminatation strategy suggested was healthcare team structures, such as one-to-five networks.

“In each unit, there is a network called one-to-five network. This is an arrangement where workers are grouped to discuss on different issues at work. So, this platform may be utilized for the introduction of the current guideline.”

(KI P6)

Fourthly, participants suggested peer education as a mechanism of dissemination and implementation of the current guideline. The fifth strategy suggested for guideline dissemination was to use unit heads as gate keepers so that the unit heads can disseminate the guideline to their subordinates. In addition, it was reported that focal persons and health professionals who work on HIV could act as role models to influence other HCWs for the implementation of the guideline as they have adequate knowledge and experience in services related to HIV. As reported by participants, existing training programs may also be utilized as an opportunity to introduce the current guideline. Suggested dissemination strategies are summarized in Table 2.

**Table 2:** Suggested dissemination strategies.

Subthemes	Categories
Active dissemination	Short-term training
	Peer education
	Workshops
	Posters at service delivery points
	Mentorship
	Regular health education programs
	One-to-five networks
	Using opinion leaders and unit heads as gateways
	Multidisciplinary team meetings
	Media
Passive dissemination	Distributing hard copies
	Publication
	Availing the guideline in libraries
	Availing the guideline through websites
	Introducing the guideline through official letters

### 3.1 Theme 3: Training

Mentioning that stigmatizing practices are widely observed in Jimma Medical Center, study participants recommended that training, mentoring and supervision be conducted for the successful implementation of the guideline. Participants reported that currently, the FMOH follows a cascaded training of trainers (ToT) when introducing new guidelines to health professionals. In addition, a one- or a two-days workshop is arranged for introducing guidelines to health managers. The paricipants also mentioned that there are regular training programs provided by HIV Prevention and Control Office (HAPCO). These training programs include orientation of health professionals on new and updated guidelines. They suggested for similar arrangements to be made for the current guideline. Moreover, they recommended an alternative in which training programs are coordinated through JMC.

Two options were suggested to cascade the training on the current guideline. One method was to train the heads of units so that they will disseminate it to their subordinates through different gateways such as mentorship and one-to-five networks. The other method suggested was first to train unit heads, then their staff in subsequent rounds.

On the other hand, two approaches were suggested for the arrangement of the training on the guideline; either to prepare a new training program or to integrate the training on the guideline into the current programs such as ART training programs. Further exploration indicated that the provision of a separate training program for the current guideline can potentially increase the attention given towards its implementation compared to integrating it with other training programs. Regarding the timing of the training, participants indicated that previous training programs are being conducted in shifts (rounds) and the same method should be utilized for the current guideline.

For the current guideline, participants categorized HCWs into two types: those HCWs who directly engage in the care and support of PLHIV and those professionals who do not have frequent direct interaction with PLHIV. They proposed the training to be provided to both categories of HCWs. They suggested a short-term training for those HCWs who directly engage in delivering service to PLHIV stating that they may not recognize their own stigmatizing actions and attitudes.

“I can mention two types of healthcare providers here. The first group is a group directly engage in delivering service to HIV positive clients. It is possible to provide a short-term training to this group. This is important because even if they are working on the area, they may not recognize their own stigmatizing actions and attitudes. The second group is a group of healthcare providers who are not directly involved in the provision of care and support for HIV positive clients. Still, they have a chance to provide the service for the clients in one way or another. It is also essential to orient these professionals through short term training on the impact of stigma. In addition, it is essential that other non-health professionals are also trained.”

(KI P1)

Some participants suggested that preparation of the training program in different formats for professionals providing care and support to PLHIV and for other health professionals is cost effective. However, all participants agreed that the guideline and the training format for all disciplines of health, medical and allied health professionals should be uniform provided that there are no budget constraints for such an arrangement. Participants also stressed the importance of mixing professionals of different disciplines and professionals working in different units to facilitate experience sharing. Regarding whether there is a need to tailor the guideline to the educational level of health professionals, it was suggested that the educational status of health professionals cannot be an obstacle for the implementation of the guideline.

### 4.1 Theme 4: Implementation issues

The key implementation issues pinpointed by the participants included the importance of encouraging partnership, advocacy and teamwork, using position holders and opinion leaders as role models, the need for implementation structure, and posting reminders.

First, partnership between stakeholders was identified as one of the central aspects in the implementation of the guideline. Participants reported that partnership resulted in the success of other programs. Participants reported that previous initiatives that tried to introduce new guidelines that have got support from stakeholders faced fewer challenges. They emphasized the need to collaborate with different stakeholders. Participants also reported that advocacy helps to get attention of decision and policy makers at different levels including zonal, regional and federal levels.

Secondly, strengthening teamwork was mentioned as a facilitating factor for guideline implementation. The negative attitudes of some professionals and communication barriers were mentioned as major barriers towards teamwork. Participants also reported that communication gaps between HCWs result in limited awareness of their roles and responsibilities which ultimately causes conflicts. And this will negatively affect teamwork. Encouraging effective communications and delineating the roles and responsibilities of different categories of health professionals were recommended as remedies to tackle barriers to teamwork.

Partcipants also identified opportunities that encourage teamwork in JMC. These include: the existence of teamwork guideline, one-to-five network, peer education and MDT meetings. In MDT meetings, health professionals share the challenges they face in their routine activities and discuss cases and learn from one another. Participants suggested that these opportunities should be utilized to improve teamwork and group learning among HCWs and to inform the HCWs about the importance of providing client-oriented respectful care. They also reported that unit heads and opinion leaders play a substantial role in building and maintaining team spirit and in strengthening the implementation of the guideline by acting as role models. As mentioned by participants, problems in team work occur when heads of units and senior staff are not involved in the agenda.

Thirdly, participants suggested that an implementation structure comprising an implementation committee and a focal person who can oversee the implementation is needed. They also suggested that the roles and responsibilities of everyone in the committee should be defined. The other method suggested for increasing adherence to the guideline was using posters for stigma mitigation and reminders in each room of the healthcare facilities. One participant mentioned an experience of using posters to remind HCWs to adhere to guidelines.

“For example, as part of increasing adherence to HIV and nutrition guideline, we have used posts that indicate body mass index (BMI) cut off points. They identify the BMI levels as green, red and yellow. This has increased adherence to the guideline. We can use the same strategy for stigma reduction guideline.”

(KI P5)

### 5.1 Theme 5: Monitoring and evaluation

The success of M&E of the implementation of the current guideline depends not only on the type and quality of data collected but also on the availability of data for M&E. As participants mentioned, this will be possible only if the data related to SAD reduction is linked to institutional Health Management Information System (HMIS) data. On the other hand, participants reported that there is a weakness both in data generation and utilization. Specially, they mentioned that currently data collected on care and support of PLHIV is not being used for decision-making.

“Last time, I had an opportunity to attend the presentation on HIV service-related report. My perception was that HIV data is complete. But, what I discovered from the presentation was that it is not being used for decision-making. The data is not well organized. There is no one who analyzes and presents the data for decision makers. There are also some data that are not being recorded. So, there is weakness both in data generation and utilization. Maybe it’s use has been weakened by the HMIS [which is used throughout the hospital].”

(KI P7)

Training, implementation, monitoring, evaluation, integration and sustainability related to the guideline are summarized in Table 3.

**Table 3:** Training, implementation, monitoring and evaluation.

Broader themes	Subthemes	Categories
Training	Current training	Workshops to create awareness among managers
		Cascading programs through ToT
	Opportunities for training	Suitable training venues in the hospital
		Committed stakeholders
		The presence of committed staff
	Suggested training strategy	Cascading through unit heads
		Cascading through ToT
	Suggested training format	Integrate into existing training program
		Prepare a new training program
		Training approaches for HCWs based on their level of contact with PLHIV
		Mixing professionals of different disciplines
		Describing the roles of each professional
Implementation	Encouraging internal and external partnership	Role of partners in success of guideline implementation
		Attention given to partnership
		Partnership aids to tackle barriers
	Strengthening teamwork	Barriers to teamwork (the negative attitude of HCWs and communication barriers)
		Remedies to tackle barriers to teamwork (encouraging effective communication and delineating the rights and responsibilities of different categories of HCWs)
		Utilizing facilitators of teamwork (one-to-five network, peer education, MDT meetings)
		The role of unit heads and opinion leaders in building team sprit
	Using position holders and opinion leaders as role models	Unit heads as potential role models
		Senior professionals as potential role models
		Opinion leaders as potential role models
	Advocacy	Advocacy for influencing resource allocation
		Advocacy as a means of dissemination
	The need for an implementation structure	The need for an implementation committee
		Delineating the roles and responsibilities of implementation committee
		The need for implementation focal person
	Posting reminders and posters	
Monitoring and evaluation	HIV-specific M&E	Frequency of evaluation
		Type of service being evaluated
		Responsible body for M&E
		Type of data being generated
		Problems related to M&E
		Limited data available in a usable format
		Staff responsible for M&E
		Type of data being collected
		Availability of data
	Current responsible body for evaluation	External evaluation
		Internal evaluation

**NB**: ToT: Training of trainers, HCWs: Healthcare workers, PLHIV: People Living with HIV, MDT: multidisciplinary team, HIV: Human immunodeficieicny virus, M&E: Monitoring and Evaluation.

### 6.1 Theme 6: Resource implications

Study participants reported that financial resources are needed to conduct training on the guideline, prepare training curriculum and manuals, to disseminate and to implement the guideline and to conduct M&E related to the guideline implementation. They reported that as part of dissemination, resources are needed to make the guideline available online or to avail the guideline and checklists in different formats and in different units. Resources are also needed to use media to popularizethe guideline and to conduct dissemination workshops.

“We need audiovisual materials to promote the implementation of the guideline. This may include videos, leaf lets, brochures or posters. It is essential to use these materials. We need these materials for the entire hospital community. For instance, we may present life history of stigma victims and the impact of stigma on clients.”

(KI P2)

Participants also indicated that materials for standard precaution should be supplied regularly. The shortages of supplies for personal protection may impede the implementation of the guideline.

“The thing is, if there is shortage of supplies such as gloves, the health professional should not perform invasive procedures for all patients not just for PLHIV. If there is a shortage of supply, the healthcare worker adhering to standard practice may refuse treating patients and his actions may be misinterpreted as being negligent. So, finally, he [the provider] may think that he was misunderstood just because of his adherence to the guideline. This may lead HCWs to conveying wrong messages and use of differential precaution, which is one component of discrimination.”

(KI P7)

In addition, participants reported that the M&E of the implementation of the guideline needs resources in the form of per diem for mentors and supervisors. However, they also reported that mentorship, and M&E may be conducted along with existing programs and hence may not require additional resources.

### 7.1 Theme 7: Integration of the current guideline into the hospital monitoring and evaluation system

Participants also identified other preconditions for the integration of the guideline into the current M&E system. These preconditions are: deciding the responsible body that owns the evaluation program and indicating the responsibilities of different stakeholders and the frequency of evaluation. As reported by the participants, it is the responsibility of the quality office of the hospital to carry out internal evaluation. They also suggested that Jimma Universty HIV Prevention and Control (JUHAPCO) should take the role as an external evaluator and the HIV and Tuberculosis Clinic should take the role of internal evaluator. They stressed that both JUHAPCO and the HIV and TB Clinic should provide reports on activities done related to stigma and discrimination to the planning office of Jimma Medical Center (JMC). For such integration to be realized, participants suggested that the indicators of the current guideline should be included in institution-level indicators, such as mentoring checklists, key performance indicators and HMIS. They reported that there is a uniform reporting system enabled through indicators developed for reporting to regional state health bureau and the MOH. They also reported that JMC can modify indicators developed for institutional level reporting.

Participants stressed that there should be mentoring and supervisory visits to monitor and evaluate the implementation of the guideline. They also reported that mentoring can be used as a platform for introducing, implementing and evaluating the guideline. Additionally, they suggested that there should be a focal person from the HIV and Tuberculosis Clinic itself who oversees the work and who closely supervises it.

Moreover, two options were proposed regarding the integration of the guideline indicators. One option was to keep the indicators separately to seek attention and give more focus for it. The other option was to integrate them into ART service evaluation or HIV services evaluation performance indicators.

Study participants reported that there is Site Improvement through Monitoring (SIM) system in JMC which they described as a system in which performance is evaluated and graded in red, yellow, amber and green colors. They emphasized that data generation on guideline implementation should not only be for the sake of simple external evaluation, but also for service improvement. The data generated should be utilized by unit managers and service providers to improve performance. Nevertheless, they admitted that currently there are weaknesses related to the utilization of data for service improvement. They suggested that the management should make a request for data and should enforce the HMIS focal person to improve data handling process.

“Managers should enforce personnel working on HMIS so that they generate appropriate data for decision-making. If they need training, appropriate training must be provided to them. In addition, the management should request for data. If there is no one in need of the data, the HMIS persons will not handle or report the data appropriately.”

(KI P7)

### 8.1 Theme 8: Scaling up and sustainability

Participants reported that currently, gaps exist in implementation and scaling up of guidelines. They suggested that the guideline should be scaled up through the provision of training of trainers for unit heads and for few staff. Describing the challenge associated with the provision of the training for all staff at the same time, they recommended that the training should be provided in rounds. Moreover, they suggested that the guideline should be integrated into a pre-service teaching curriculum for allied health, medical and health science students.

**Fig 1:**
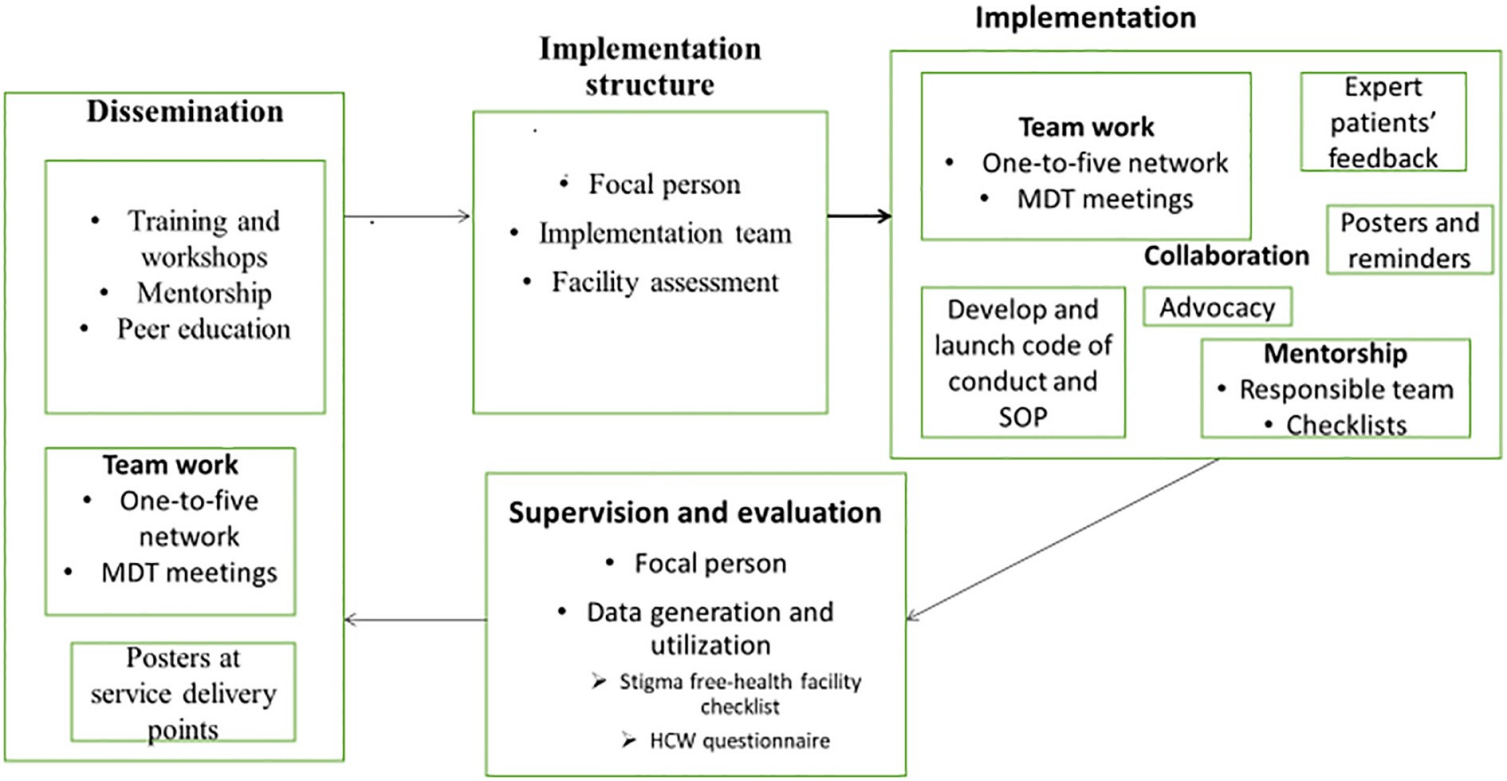
Suggested implementation procedure for the guideline.

Key informants recommended that the guideline should be scaled up nationwide after piloting in JMC and collecting data on all the challenges related to the implementation. They also added that workshops and conferences should be prepared to introduce the guideline to stakeholders at regional and national levels. The themes generated under resource implications, integration, scaling up and sustainability are summarized in Table 4. Based on the findings, we have suggested a framework comprising dissemination, training, implementation and evaluation components (Fig 1).

**Table 4:** Resource implementation, integration, sustainability and scale up.

Broader themes	Subthemes	Categories
Resource implications	Resources for training	Per diem for trainers and trainee
		Preparation of modules and manuals
		Printing posters, guidelines and handbooks
	Resources for dissemination	Printing the guideline
		Publishing
		Arranging media
	Resources for implementation	Facilities for standard precaution
		Resource for monitoring, supervising and mentoring
Integration	Data collection for M&E	The need to create a culture of utilizing data to improve performance
		Site improvement though monitoring system (SIM)
	Tools and checklists	Mentoring checklists
		M&E checklists
	Integrating the guideline with mentorship and supervisory visits	Mentorship as dissemination strategy
		Mentorship to provide an onsite technical support during implementation
		Mentorship for the evaluation of adherence to the guideline.
		Integrating checklists related with stigma into mentoring checklists
	Suggested responsible body for supervision and evaluation	Experienced professionals
		A professional who has been trained on the guideline
		The need for internal focal person for evaluation
		Need for an outside evaluator
		The need to enforce and train personnel working on HMIS
Scaling up and sustainability	Platform for sharing best practice implementation experience	Professional conferences
		Workshop for policy makers
	Initial small-scale implementation at JMC	Collecting data on implementation experience

**NB**: HMIS: Health Manangement Infromation Sytem, JMC: Jimma Medical Centre.

## Discussion

The current research identified the following broad themes: barriers and facilitators, dissemination approaches, training, implementation, monitoring and evaluation (M&E), resource implications, integration of the guideline into the hospital routine, sustainability and scaling-up.

### Barriers and facilitators

One of the major themes identified in the current project is barriers and facilitators to implement the guideline. In their theoretical framework for theory informed behavior change interventions to implement evidence, French et al.[33] emphasized the need for the identification of barriers and facilitators. They specifically noted the value of identifying modifiable barriers and specific roles of stakeholders to address the barriers. As we have already outlined in the results section, most of the factors identified in the current study can be both facilitators and barriers for the implementation of the guideline. Similarly, previous researchers have identifed factors acting both as facilitators and barriers to the implementation of guidelines [34, 35]. Therefore, we have presented barriers and facilitators together. Such a presentation format has been used by previous researchers [34, 35]. In the current project, we identified three subthemes of facilitators and barriers: characteristics of the guideline, the practice setting and provider-related factors.

Scholars recommend using a theoretical framework to systematically identify and address factors that impede guideline implementation [33, 36, 37]. David et al.[38] categorized factors affecting implementation of innovations and guidelines into six domains, namely characteristics of the guideline, characteristics of the health professionals, the practice setting, incentives, regulations and patient-related factors [38]. In line with this, the current project identified barriers and facilitators related to these six factors as follows:

**Characteristics of the guideline**. In the current research, we identified the following factors inherent to the guideline that potentially impact the uptake of the guideline prepared to reduce HIV-related SAD: clarity, comprehensiveness, compatibility with existing practice, initiatives and system, all of which were facilitating factors in the context of the study area. Previous research has indicated that the lack of trialability, compatibility and observability, and complexity of guidelines may deter the implementation of guidelines [38, 39]. On the other hand, for the current guideline, trialability was identified as a facilitating factor if training is provided for HCWs. In addition, the existence of up-to-date recommendations in the guideline was identified as one of the good qualities of the current guideline facilitating its uptake.
  The potential positive impact of a guideline on the clinical process facilitates the uptake of the guideline [35, 40]. On the other hand, the lack of expectation of the desirable outcomes of adherence to a guideline may hinder the implementation of the guideline [18, 40]. For the current guideline, participantsreported that the reduction of SAD contributes not only to the success of HIV-related goals, but also to other initiatives, such as service quality improvement, CASH and CRC initiatives.
**b) Attributes of health professionals (provider-related factors)**. The awareness and motivation of HCWs facilitates the uptake of a guideline [35, 40]. In our study, the motivation of HCWs, especially those working in the HIV and Tuberculosis Clinics, was identified as a facilitating factor for the dissemination and implementation of the current guideline.
  On the other hand, participants reported that provider-related factors such as unfavorable provider attitude and lack of awareness aboutthe guideline negatively impact the implementability of the guideline. Unrealistic expectations or limited awareness of the guideline among HCWs potentially hinder the uptake of the current guideline. In agreement with this, previous research reported that the lack of awareness of the existence of the guideline and limited familiarity with the content of the guidelines or disagreement with the recommendations may negatively affect the implementation of guidelines [35]. As a remedy for this, study participants suggested that the guideline should be disseminated through existing opportunities and platforms such as MDT meetings, one-to-five networks, and training and mentorship programs.
  To ensure use of evidence by healthcare providers, it should be tailored to local context [15, 19]. In the stigma reduction guideline, we tried to build the sense of ownership among local stakeholders and tailored the guideline to the local context. Similarly, previous researchers indicated that guidelines developed by end-users or by consensus methods increased clinicians’ ownership of the guideline and were associated with increased compliance [41]. In addition, the involvement of health professionals from local institutions in designing, implementation and dissemination strategies facilitates the uptake of a guideline [41].
**c) The practice setting (organizational policy and practice related factors)**. In this project, we identified factors in the policy and practice environment (practice settings) that affect the implementation of the guideline. The implementation of a guideline depends on the ability of multiple stakeholders to plan and execute the various steps needed to implement the guideline [42]. Global evidence indicates that the lack of management support hampers guideline implementability [18, 40]. For the current guideline, as reported by study participants, the management of Jimma University and the JMC is committed to support and facilitate the implementation of the guideline as it contributes to priority goals of the hospital, improving quality of health services.
  In addition, the existence of training programs and venues and committed stakeholders were identified as facilitators. In support of this, previous SAD reduction guidelines emphasized the necessity of convincing stakeholders during the implementation of SAD reduction programs [43].
  Organizational factors such as resource limitations may hamper the implementation of guidelines [18]. For the SAD reduction guideline, the continuous supply of materials for standard precaution demands someresources. The current study revealed that JMC is committed to providing these materials continuously. In the long run, however, SAD reduction will contribute to the reduction of extra-precaution which will in turn reduce unnecessary wastage of resources.
  Work overload is one of the factors that commonly impede adherence to guidelines [18, 40]. The same concern was raised in the current project. On the other hand, stigma reduction, as study participants reported, in the long run can contribute to the reduction of unnecessary work load that results from bypassing nearby facilities to seek care at facilities in large towns and cities. This is related to patients seeking healthcare from facilities that are far from their locality to hide their sero-status from their neighbors in fear of stigma and discrimination.
**d) Incentives**. In the current project, factors related to incentives were already addressed under provider-related factors. Among the provider-related factors expected to hinder the implementation of the current guideline were unrealistic expectations of incentives during training and implementation of the guideline. However, HCWs directly involved in the care and support of PLHIV are relatively better compensated compared to other HCWs. This may positively affect the uptake of the guideline at the study hospital. Previous researchers have reported that limited structural support such as financial disincentives may negatively affect the implementation of a guideline [18, 40].
**e) Regulations**. The regulation of guideline implementation by accreditation or licensing bodies facilitates the implementation of a guideline [38]. Currently, there are mentoring, monitoring and evaluation systems in the Ethiopian context that are relatively stronger in HIV-related practices. The fact that stigma is a human rights issue [44] was raised as a facilitator for the implementation of the current guideline. The current study also indicated that, as one of the guidelines address ethical and governance issues, there is an opportunity for better uptake of the guideline. Therefore, it is possible to integrate SAD reduction guideline into the regulation, monitoring and evaluation systems of healthcare facilities. Moreover, participants suggested that the guideline should be used as a teaching material for allied health, medical and health science students, in which case it will also be incorporated as part of the professional accreditation system.
**f) Patient-related factors**. The presence of empowered and educated patients that ask for the right information and demand for standard practice facilitates the uptake of a guideline [38]. The existence of ‘expert patients’ (HIV positive clients who are used as supporters to their fellow patients) was presented as a facilitator for the empowerment of other patients. In addition, the guideline informs the rights and responsibilities of clients empowering them with adequate information.
  Similar to what has been elaborated above, according to the framework suggested by RNAO [26], the expected barriers and facilitators for the implementation of best practice guidelines can generally be categorized into: evidence (guideline) related factors, target audience (provider) related factors, and organizational context (practice settings) in which the guideline is to be implemented and resources needed for the implementation [26]. Most of these components overlap with the framework suggested by David et al.,[38] but regulation and incentive components were not emphasized by RNAO [26]. As described above, the factors identified as barriers and facilitators can be conceptualized using the conceptual framework developed by Dave et al. [38] and RNAO [26].

### Dissemination

When implementing new guidelines or improving adherence to guidelines, one of the practical challenges is bringing about change in HCW’s behavior. Drawing on the diffusion of innovation theory, trans-theoretical model of behavior change, health education theory, social influence theory, and social ecology, and evidence from systematic literature reviews on the effectiveness of behavior change strategies, Moulding et al. [45] developed a dissemination and implementation framework for guidelines. Their framework underscored that there is a need to assess the readiness of practitioners for the implementation of guidelines, of barriers to change and the levels at which the interventions should be targeted [45]. The current project sought the readiness and commitment of relevant stakeholders, including health professionals to implement the newly developed guideline.

In their review reported in 2013, McCormack et al. [46] found that multi-component dissemination strategies are more effective at improving guideline adherence compared to a single dissemination approach. However, there is no sufficient evidence to recommend one method over the other. Though different dissemination mechanisms have been used by policy makers and guideline developers, preferable methods depend on local circumstances [46]. In the current project, though different alternatives were suggested, no specific combinations were suggested for dissemination. However, study participants categorized the dissemination strategies into active and passive methods. Orientation workshops, training, one-to-five networks, MDT meetings of HCWs and mentorship programs were suggested as preferable and active mechanisms of dissemination. On the other hand, distribution of hard copies, publishing and availing the guideline in libraries and websites were identified as passive mechanisms of dissemination, but as potential strategies to substantiate other mechanisms. In agreement with these findings, Grimshaw et al. reported that there is moderate quality of evidence indicating that the distribution of educational materials to HCWs improves patient outcomes [47].

In agreement with our findings, Prior et al. [41] reported that multifaceted interventions, interactive education and clinical reminder systems are effective guideline implementation strategies. On the other hand, they reported that passive education and information dissemination methods such as conferences, websites and didactic lectures were not effective in guideline implementation [41]. Additionally, other researchers have identified factors that enhance the implementation of guidelines such as reviewing, reporting and publishing guidelines [48]. In the current study, participants recommended distribution of the guideline both in hard and soft copies to enhance the dissemination effort. The dissemination mechanisms that are effective in one setting may not work in other settings. Although some health professionals in the current study context have access to computers, not all of them do. In addition, internet availability is limited. Therefore, preference of active dissemination strategies over passive ones is logical.

### Implementation

Guideline development organizations utilize different mechanisms to promote the implementation of guidelines. These include: online reminders, educational outreach, interactive educational techniques and multifaceted interventions [49]. In the current study, the suggested mechanisms for effective implementation were provision of short-term training and workshops, using posters at service delivery points, using expert patients and integrating the guideline into mentorship and MDT meetings. In agreement with this, Grimshaw et al. found a moderate quality of evidence indicating that reminders lead to improvement in patient care [47].

Participatory educational interventions increase the uptake of guidelines by end users [45]. Similarly, in the current project, participants reported the uptake of the guideline can be potentially improved through training and mentorship programs. Practice facilitation is among the mechanisms for enhancing the implementation of practice guidelines [50]. Baskerville et al. [51] found that primary care facilitators were more likely to adopt evidence-based guidelines through practice facilitation. In line with this, participants of the current project suggested mentorship as a mechanism for dissemination, implementation, monitoring and evaluation of the current guideline. Furthermore, in the field of HIV, an earlier research showed that mentorship programs have been successful in facilitating the dissemination of new and evidence-based practices [52].

Assigning leaders was recommended as a critical step inthe implementation of a guidelineby the Scottish Intercollegiate Guideline Network [49]. We also found that having clear implementation structure with a focal person assignedis essential for effective implementation of the proposed guideline. It has also been reported that the accuracy and timeliness of organizational and inter-organizational information systems affect the implementation of a guideline [42]. In the current project, participants suggested that guideline indicators should be integrated into the existing monitoring and evaluation system of the hospital. In addition, participants identified delineating roles and responsibilities and strengthening teamwork among the necessary factors for the implementation of the guideline. As suggested by the participants of the current project, partnership within and outside the organization can also be utilized to tackle barriers during the implementation of the current guideline. One-to-five networks and MDT meetings of HCWs were suggested as gateways for dissemination, implementation and evaluation of the guideline. In line with this, Grimshaw et al. [47] reported that there is low-quality evidence indicating that educational meetings improve patient care [47]. The existence of ‘expert patients’ in JMC was suggested as a potential gateway to enhance adherence to the current guideline. Similarly, Grimshaw et al. [47] found moderate quality evidence supporting patient-mediated interventions to improve professional performance.

The integration of a guideline into routine records increases the uptake of, or the adoption of the guideline [45]. In the current project, we found continuous monitoring, evaluation and mentorship programs as critical elements in the integration of the guideline into the system of the hospital. Study participants recommended that checklists and monitoring and evaluation tools should be integrated into mentorship, HMIS and key performance indicators of the hospital. In line with these findings, Grimshaw et al. found a moderate quality of evidence indicating that putting audit and feedback systems in place improves patient care [47].

While the current study identified potential barriers and facilitators to implement the newly developed guideline, it was limited to the context of Jimma Medical Center although participants have also raised important points relevant to most healthcare institutions in Ethiopia. This calls for caution when generalizing the findings reported here to the context of other health facilities.

## Conclusion

In the current project, we sought experts’ perceptions both on the facilitators and barriers to the implementation of the guideline on the reduction of SAD related to HIV. We identified factors related to the nature of the guideline, the policy and practice environment, the health professionals and the commitment of stakeholders that potentially impact the uptake of the guideline. Policy makers and health managers should take note of the barriers and facilitators reported in here to effectively introduce and implement the guideline on the reduction of SAD related to HIV. Organizing tailored trainings and workshops to popularize the guideline among healthcare providers was suggested. Mentorship programs, MDT meetings and one-to-five networks can be used as a mechanism of dissemination and implementation ofthe current guideline. Teamwork and partnership with stakeholders in and outside the hospital should be strengthened to tackle barriers related to the implementation of the guideline. In addition, it is essential to establish an implementation structure comprising an implementation committee and a focal person in each health facility.

The indicators for stigma reduction guideline should be integrated into mentorship, MDT meetings and evaluation programs of the hospital. Facility managers and unit heads should make sure that the data collected for M&E is being utilized to improve performance. Moreover, as the guideline is being implemented in JMC, data on implementation experiences should be collected to assist decision about the scale up of the guideline throughout the country and beyond.

## Supporting information

S1 Doc Semi structured interview guide. (DOCX)

## Abbreviations

AIDS: Acquired Immune-Deficiency Syndrome
CASH: Clean and Safe Health Facility
CRC: Compassionate, respectful, and caring
HIV: Human Immunodeficiency Virus
JBI: The Joanna Briggs Institute
JMC: Jimma Medical Centre
JUHAPCO: Jimma University HIV Prevention and Control Office
M&E: Monitoring and Evaluation
MDT: Multidisciplinary team
PLHIV: People Living with HIV
RNAO: Registered Nurses Association of Ontario
SAD: Stigma and discrimination
SIM: Site Improvement through Monitoring
UN: United Nations
