# Supplementary material for: Exploration of facilitators and barriers to the implementation of a guideline to reduce HIV-related stigma and discrimination in the Ethiopian healthcare settings: A descriptive qualitative study
Source: PLoS One. 2019 May 13;14(5):e0216887. doi: 10.1371/journal.pone.0216887 (PMC6513051; doi:10.1371/journal.pone.0216887)
Supplement: S1 Doc — (DOCX) [file pone.0216887.s001.docx]

# S1 Doc: Semi-structured interview guide for health professionals and health managers

**The University of Adelaide,**

**Faculty of Health Sciences**

**School of Public Health, Joanna Briggs Institute**

**Semi-structured interview guide**

**Introduction**

A guideline working group has developed an evidence-informed guideline for reducing HIV-related stigma and discrimination in healthcare settings. This guideline represents an appropriate means to reduce HIV-related stigma and improve quality of services delivered to people living with, and affected by HIV.

Before disseminating the guideline in the form of publications and conference presentations, we would like to assess facilitators and barriers for the implementation of the guideline recommendations in Ethiopian healthcare settings. As a health, professional/health manger working with HIV patients, we believe that you will provide us some information on barriers and facilitators towards the implementation of the guideline recommendations. Our interview may take 30 min to one hour. Is the environment where you are sitting right now suitable? Do you need to make more adjustments so that there will be no disturbance during our discussion? If you are comfortable, we can continue our discussion now.

1. **Questions related to the nature of the evidence**
2. Have you read the document (guideline)?
3. Are the recommendations included in this guideline clear and easy to understand? If not, how might you make them so?
4. How might the guideline be made accessible to health professionals and health managers (facility heads, department heads, zonal and district health department heads)? Can it be built into current documentations? If not, what modification should be made?
5. How can the recommendations included in this guideline be implemented?
6. What might prompt the implementation of the guideline recommendations? What are the obstacles and facilitating factors for the implementation?
7. Can you give us information about tools related the current guideline?

Probe: Are additional tools needed? Are the tools suggested clear and easy? If not what type of tools should be added?

**B. Questions related to potential adopters**

1. Are the schedules of health professionals flexible to allow them attendance at meetings and education sessions? If not, what arrangement could be made?
2. Have health professionals in your facility ever been exposed to evidence-based practice? If so, what has their previous experiences been? What went well? What did not go well? What can we learn from previous experiences? How can we modify the implementation approach?
3. Do health professionals in your department work together as a team? How might that team work be improved?
4. Do department heads or senior health professionals play role model in implementing the new recommendations?
5. How is staff education on new protocols and guidelines delivered currently? How well does it work? If it works could the education needed to implement the current guideline recommendations be provided in this format? If not, is there another approach to education that might work with health professionals in your unit?
6. Is there any specific format that you recommend for the category of health professionals? (Nurses, health officers, medical doctors, clinical psychologists, psychiatrists, laboratory technicians, etc.). If so, describe it.

**C. Resources**

1. What resource constraints do you expect? (Probe: time, human, material, financial)? How can these resource constraints be tackled for the implementation of the guideline recommendations?
2. **Environmental factors**
3. What other competing interests of the organization impede the implementation of the recommendations?
4. What are the corporate priorities? Does the implementation of these guideline recommendations complement the strategic goals for HIV control and prevention? What impact does it have on infection preventions and patient safety?
5. What is the patient load of the facility? Does this impede the implementation of the recommendations?
6. Are there adequate facilities for meetings and educational sessions (rooms, time, and motivated staff)?
7. **Audit and evaluation**
8. What types of data are already being monitored in the organization (hospital and JU and Jimma zone HAPCO)? Can you obtain access to that data?
9. Is there any regular audit for practice in this health facility? Describe in detail.
10. Has ever current practice been evaluated? Describe activities evaluated.
11. Will evaluation require further resource support? If so what type pof resources? Who can do the audit?
